# Unveiling the potential of digital twins in homecare: A reflexive thematic analysis of older adults’ views

**DOI:** 10.1177/20552076261450290

**Published:** 2026-05-12

**Authors:** Sandra Saade, Susanna Nordin, Kevin McKee, Marie Elf, Johan Borg

**Affiliations:** 1 School of Health and Welfare, 101092 Dalarna University, Falun, Sweden; 2 Post Graduate School for Integrated Care, Örebro University, Örebro, Sweden

**Keywords:** digital health, homecare, integrated care, older adults, person-centred care

## Abstract

**Objectives:**

A growing number of frail older adults live at home, placing increasing demands on health and social care systems. Digital twins may improve homecare services and support person-centred and integrated care. This study investigated older adults’ experiences of homecare and their perspectives on the potential use of digital twins in homecare.

**Methods:**

An exploratory, two-stage qualitative study was conducted with 14 older adults from two Swedish municipalities. In the first stage, semi-structured interviews explored participants’ experiences of homecare. In the second stage, the same participants took part in either a semi-structured interview or a focus group discussion regarding their views on digital twins, with participants choosing their preferred format. Reflexive thematic analysis was used to interpret the data.

**Results:**

The analysis generated one main theme, Navigating uncertainty and possibility in digital twin-supported homecare, with four sub-themes, Challenges in understanding and accepting digital twins, Concerns about privacy and ethics, Opportunities for safety and prevention, and Potential effects on daily routines. Participants struggled to comprehend the nature of digital twins. Concerns about their potential use in homecare included ethical issues, impacts on privacy, and reduced social interaction with homecare staff. However, participants also identified potential benefits of digital twins, such as more responsive services, improved scheduling, and enhanced safety through monitoring and predictive functions.

**Conclusion:**

This study highlights the potential of digital twins in homecare from the perspective of older adults. While digital twins offer opportunities, integrating them into homecare services is complex and requires addressing the concerns raised by participants.

## 1 Introduction

The social policy of ageing in place, which promotes older adults’ continued residence in their own homes,^1^ aligns with many individuals’ preferences^2–4^, and is associated with enhanced quality of life, independence, well-being, and privacy.^5^ However, this policy may also have unwanted consequences, such as social isolation,^6^ and some homes may be in poor condition, lacking the necessary support for ageing well.^7^ The global increase in the older adult population,^8^ has led to a rise in older adults with complex health conditions over the past two decades,^9^ many of whom, due to the ageing in place policy, continue to live at home.^10^ Consequently, homecare users have become more frail and experience poorer health.^11,12^ The ageing population is also resulting in an increase in informal carers,^13^ who provide most of the care received by older adults across OECD countries. However, these carers often experience mental health strain^14^ and have unmet needs, including a need for support.^15^ The expenses associated with services that support daily living can place a significant financial strain on older adults,^16^ as well as on social and healthcare services, due to rising demand and ongoing recruitment challenges.^17–19^ In light of this, the potential of digital technology for addressing the health and social care needs of older adults is being explored.^20^ This paper reports a Swedish study of older adults’ perspectives on a specific type of digital health technology, a digital twin, and its potential for use in homecare.

In recent years, digital twins have gained research attention and demonstrated proven utility in healthcare settings.^21^ A digital twin is a virtual model of a person, a system, a space, or some other physical entity with a connection between the virtual model and the physical entity that enables data transfer in both directions.^22^ Digital twins can be used for several purposes. Digital twins of cells, organs, and whole bodies are used for medical purposes, and are possible solutions for personalized diagnosis and treatment of diseases.^23–25^ Digital twins can enhance care and well-being for individuals by monitoring their behaviour and predicting outcomes. They can also be used to create more suitable care environments, with better management, optimization, planning, and coordination.^21,26,27^ Digital twins offer the opportunity to provide personalized and proactive care,^26–29^ and have been tested in home environments to detect falls, to predict disease,^30^ and to secure the homes^31^ with positive results. One study used digital twin technology based on daily activities, sleep patterns, environmental conditions, and time spent outside the home to detect potential health risks, and the results showed that the system is feasible for providing real-time insights, alerts, and can support older adults to age in place.^32^ Although digital twins hold significant promise, several challenges remain unresolved, including technical infrastructure, implementation costs, and ethical issues.^22^ Moreover, research on the application of digital twins in the homecare context is lacking.^33^ User acceptability is not well understood,^34,35^ and the perspectives of older adults, essential for guiding user-friendly design and ensuring that technologies are responsive to their needs, remain underexplored.^36,37^

Homecare services should be tailored to the capacity and needs of every person and address physical, mental, and social factors.^38^ In Sweden, 11.7 percent of women and 8.4 percent of men over the age of 65 received some form of homecare services in 2024, with the proportion increasing with age.^39^ The Swedish Social Service Act^40^ states that homecare services for older adults should promote an active lifestyle and participation in daily activities. Care must be of good quality, be individualized, respect every person’s privacy, integrity, and self-determination, and allow the older adult to make their own choices according to when and how services are given. Services should also focus on interventions that are sustainable in the long term and grounded in preventive and health-promoting approaches.^40^ Sweden’s recent integrated care reform^41^ emphasizes person-centred approaches to health and social care, consistent with internationally accepted frameworks for person-centred and integrated care. A digital health strategy is regarded as integral for implementing these frameworks.^38^

Older adults want to be seen as unique individuals, be listened to, receive the right information at the right time, and be involved in decision-making.^42^ A person-centred approach focuses on creating meaningful relationships between the providers and the persons in need of care. It highlights the importance of skilled and compassionate staff, a supportive care environment, and care practices that recognize the uniqueness, values, and preferences of each individual.^43^ There is a worldwide shift towards integrated care, aiming to shift health and social services to focus more on person-centred and coordinated care.^44^ Integrated care is often described as including a multidisciplinary team and proactive and person-centred approaches.^45,46^ Integrated care can increase older adults’ functionality and satisfaction with care, postpone relocation to a residential care facility,^46^ improve the quality of care,^47^ and decrease hospital admissions.^48,49^ A review of studies of integrated person-centred care interventions for older adults living at home in Sweden found the interventions to have their greatest effect on service utilisation (66% of interventions successful) and disability (61%), with less effect on morbidity (46%), subjective health (30%) and mortality (25%).^50^ Integrated care has been proposed as a way to strengthen health systems in response to demographic change, with a particular emphasis on prioritising community-based services.^51^

Research from various countries suggests that older adults often lack well-coordinated and integrated healthcare services^52,53^ and desire better access to homecare,^52^ while those receiving homecare can feel less independent, lose confidence in their capacities, and report little influence over their care.^54^ Reportedly, homecare services in Sweden are far from fulfilling the implementation of person-centred care and integrated care.^55,56^ Many older adults living at home do not have the opportunity to participate in their homecare and to live their lives as they wish due to a lack of information, short visits, time pressure, not getting help at the desired time, and lack of staff continuity.^57^

The success of digital twins in healthcare settings raises the question of whether such technology could have a similar impact in homecare for older adults, specifically in addressing the above shortcomings in current homecare quality and the shortfall in homecare staff. However, research on the technology’s potential value for improving homecare services is limited. Before using digital twins in homecare, older adults’ perspectives must be explored, so that implementation can be carried out in a way that is responsive to their needs and concerns. This paper describes an exploratory and user-focused Swedish study, the purpose of which was to investigate older adults’ perspectives on the potential use of digital twins in homecare, informed by their personal experiences of the service. The study’s research questions are:

• What awareness and understanding do older adults have of digital twins?
• How do older adults view the potential use of digital twins in homecare?

## 2 Methodology

### 2.1 Design

This study employed an inductive qualitative approach with data collected through semi-structured interviews and focus groups.

### 2.2 Participants

Purposive sampling was used to recruit participants who varied on characteristics theorised to be of relevance to the study aim: age, gender, type of housing, place of residence (urban/rural), and kind and frequency of homecare services. The inclusion criteria for taking part in the study were that the participant was 65 years or older and in receipt of homecare. Individuals with cognitive impairments or those unable to communicate in Swedish were excluded. To ensure broad representation, participants were recruited from one urban/rural municipality with approximately 60,000 inhabitants, and one rural municipality with approximately 11,000 inhabitants, both located in central Sweden.^58^ Before data collection, meetings were held with homecare service managers from the selected municipalities to inform them of the study’s aim and design. They then passed this information to care managers, who identified eligible individuals based on the inclusion and exclusion criteria. Interested individuals gave their consent for their contact details to be shared with the first author. The first author contacted potential participants by telephone to provide additional information and sent written information by post. All individuals contacted agreed to participate in the study. The recruitment stopped once sufficient information power (i.e., data richness and relevance^59^) was achieved, which is the preferred criterion for determining sample size in reflexive thematic analysis rather than data saturation.^60,61^ In total, 14 persons participated.

### 2.3 Procedure

**Figure 1.**
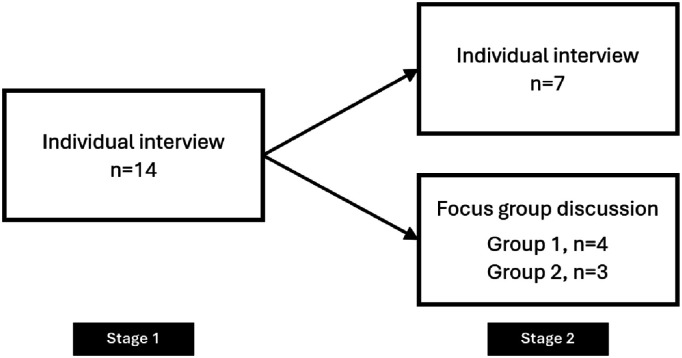
Overview of data collection.

Data collection was conducted in two stages. In the first stage, semi-structured interviews were conducted to explore participants’ views on homecare. In the second stage, the same participants were invited to take part in either a semi-structured interview or a focus group discussion regarding their views on digital twins, with the participants choosing which format they preferred. (see Figure 1).

The stage one interviews focused on participants’ backgrounds and experiences of homecare services (see Appendix 1). These interviews were designed to provide a deeper understanding of their life situations and interactions with homecare, as well as build trust between the researcher and the participants. The insights gained at this stage formed an important foundation for developing the interview/focus group guide for stage two, ensuring that the questions were grounded in the participants’ realities and relevant to their everyday challenges. These insights were also essential for understanding how participants reasoned and thought about their situations.

An interview/focus group guide for stage two was developed and pilot-tested through an interview with one older adult receiving homecare services. The purpose of the pilot interview was to assess the acceptability of the data collection procedure,^62^ and to familiarize the interviewer with the questions. Some minor revisions were made to the interview/focus group guide after piloting.

In stage two, participants were asked about their views on the use of digital twins in homecare. The first author began the interviews and focus groups by providing a brief, simplified explanation of a digital twin, including examples of sensors and measurements that could be used in the home. Open-ended questions and prompts were employed to guide the discussions (see Appendix 2). During the focus groups, the second author took field notes to capture non-verbal cues and group dynamics. The stage two interviews took place in the participants’ own homes, lasting approximately 30 minutes on average (range: 20-53). One focus group was conducted in each of the two municipalities and took place in a centrally located venue, either at the university or in a municipal venue. The focus groups lasted for 57 and 61 minutes, respectively, exclusive of a short break in the middle of the discussion.

The interviews and focus groups were conducted during the spring of 2024. One participant was interviewed in the presence of their partner. Although the partner occasionally confirmed or elaborated on the participant’s statements, the interview was directed at the participant, and only the participant’s contributions were analysed. All interviews and focus group discussions were audio-recorded and transcribed verbatim. The first author transcribed one focus group discussion, and the rest of the recordings were transcribed by a professional transcriber.

### 2.4 Data analysis

We conducted a reflexive thematic analysis following the six phases presented by Braun and Clarke (2006, 2019),^63,64^ using an inductive approach where codes and themes were generated from the data. Semantic (explicit) and latent (underlying) features of the data were considered during the analysis.

In phase one, the first and second authors familiarised themselves with the data by reading and re-reading the transcribed material and writing down ideas. In phase two, initial codes were generated by systematically going through the data. The first and second authors worked closely together throughout all steps of the analysis. The remaining study authors gave input throughout the process. In phase three, these codes were sorted into candidate themes with a core concept that connected them. In phase four, the candidate themes were reviewed and refined by going through the coded data to ensure that they reflected the content of the dataset. In phase five, the themes were defined by finding the core of the data. The themes were discussed in the research group and named appropriately. The sixth phase consisted of writing the different analysis steps transparently and clearly. The entire process was iterative, going back and forth between the original transcription, codes, and themes, with regular meetings of all researchers.

Quotations translated from Swedish to English by the authors formed part of the process of making the interpretation of the data clearer*.* The checklist of consolidated criteria for reporting qualitative research (COREQ)^65^ was used.

### 2.5 Reflexivity

The meaning in reflexive thematic analysis is constructed through the interaction between the data and the researchers’ contextual and theoretical interpretation.^60^ Therefore, throughout the analysis, we have continuously reflected on how our backgrounds, experiences, and preconceived ideas influenced the interpretation. Our background and expertise helped throughout the building of the structure of the study and the analysis process. The first author is a female doctoral student with a bachelor’s and master’s degree in nursing, while the second author is a female lecturer with a PhD in medical science and experience in ageing research and qualitative methods. Three members of the research team (females) are nurses and have worked with older adults, many with homecare services, while the remaining two members (males) have substantial research expertise in homecare for older adults, one with experience in digital health technologies. Four out of five of the researchers are skilled in using qualitative methods. During the research process, we had frequent meetings where we engaged in critical discussions. We believe these discussions enhanced our understanding of the data and supported us in the analysis and writing stages.

### 2.6 Ethical considerations

The study followed the ethical principles of the Declaration of Helsinki^66^ and was approved by the Swedish Ethical Review authority (2023-04272-01). Written informed consent was obtained from all participants.

## 3 Results

A total of 14 participants with a mean age of 88 years were included in the study. Eight participants were from the larger municipality and six from the smaller municipality. Twelve of them lived alone. The number of homecare services they had varied from three times a week to five times a day, while three participants also had services during the night. Besides the daily or weekly visits, some participants also had extra planned visits for help with showering, cleaning, and social activities. In addition, the participants had received homecare services for half a year to thirteen years. Table 1 contains information on the participants and the data collection method they selected for stage two.

**Table 1.** Participants’ characteristics and stage two data collection methods.

Participant number	Age	Gender	Type of dwelling	Frequency of homecare visits	Experience of homecare services (years)	Data collection method
P1	71	Male	Apartment	6 times/day	3	Interview
P2	90	Female	House	2 times/day	2	Focus group
P3	76	Male	House	2 times/day	10	Focus group
P4	87	Male	House	2 times/day	0.5	Focus group
P5	99	Female	House	5 times/day + 2 times/night	10	Interview
P6	89	Female	Apartment	3 times/week	3	Focus group
P7	84	Male	Apartment	4 times/day + 1 time/night	13	Interview
P8	90	Female	Apartment	3 times/week	4	Interview
P9	87	Female	Apartment	3 times/day	2	Focus group
P10	91	Female	Apartment	1 time/day	2	Focus group
P11	91	Female	Apartment	2 times/day	10	Focus group
P12	79	Male	Apartment	4 times/day	1	Interview
P13	93	Female	House	3 times/day + 2 times/night	5	Interview
P14	77	Female	House	3 times/day	1	Interview

The reflexive thematic analysis produced one main theme: *navigating uncertainty and possibility in digital twin-supported homecare* and four sub-themes: *challenges in understanding and accepting digital twins, concerns about privacy and ethics, opportunities for safety and prevention,* and *potential effects on daily routines.* Table 2 presents an overview of the main theme, sub-themes, and a description of their content.

**Table 2.** An overview of the main theme and sub-themes.

Navigating uncertainty and possibility in digital twin-supported homecare	
Challenges in understanding and accepting digital twins	*Difficulties understanding the technology of digital twins, how they could be used, and who would benefit from them.*
Concerns about privacy and ethics	*The digital twins, seen as monitoring systems, instilled discomfort, and a sense of devaluation as a human being was introduced.*
Opportunities for safety and prevention	*The potential of digital twins to enhance personal safety and security and to support preventive care and services.*
Potential effects on daily routines	*Digital twins in homecare may influence the provision of care and support in relation to older adults’ daily routines, highlighting both concerns and opportunities.*

Quotations are used to support the main theme and sub-themes, and individuals referred to as staff or members of staff are the personnel who provide homecare services.

### 3.1 Navigating uncertainty and possibility in digital twin-supported homecare

The overarching theme revolved around the uncertainty surrounding digital twins in homecare while also acknowledging the possibilities they may offer in supporting a more tailored care. In the following sub-themes, the possibilities and concerns raised by the participants are examined in greater detail.

### 3.2 Challenges in understanding and accepting digital twins

Most of the participants had difficulties in understanding what digital twins are and how they would work. They had to be given examples of what kind of information digital twins could handle or provide before they could express an opinion at all.

“It is beyond my world....I find it hard to imagine” (P5).

“How will the person who is planning know if they don't talk to us and don't see us in the picture? Maybe we're lying there and can't answer” (P2).

Since it was challenging for our participants to anticipate the potential outcomes of digital twins, it was difficult for most of them to give specific examples from their daily lives describing how they would like digital twins to be used. More often, they thought about how it could help people other than themselves. While a few participants viewed themselves as so dependent on homecare services that they could not benefit from a digital twin, others felt they were too independent to see its relevance. This latter idea reflects a perception that such technologies are more relevant for other individuals in more vulnerable situations. For instance, their responses shifted toward considering how the digital twin might be useful for those living with cognitive impairments and dementia.

“For those who are a bit demented, they wander off…then you can see if they go out. But then, will there be staff? It’s lacking sometimes as well.” (P7).

The participants also had concerns about whether the digital twin would require more resources in the form of staff and money.

“What does it cost?... and who will pay for it?...because it has to become a cost.” (P1).

### 3.3 Concerns about privacy and ethics

The initial thoughts that most participants had were that they would feel monitored by digital twins, which made them uncomfortable, and left some of them thinking about their value as human beings.

“...You are monitored to the max, I think that's the wrong way to go, should you be monitored? Is that what we are aiming for?... It’s not human at all.” (P14).

“In a way, I think it's creepy, that you're no longer a human being, you're an object.” (P2).

Some participants were anxious about homecare services being taken away from them because of a digital twin. There was a worry that digital twins could be used against them and to control them. Some participants did see a risk of being controlled by the homecare services due to information gathered through the digital twins.

“It is if they find out something and I am left without homecare services…” (P4).

“Yes, not danger but….no, I don’t know. It sounds a bit scary at the same time, I think, controlling.” (P8).

In general, the participants approached the idea of using a digital twin in their home with caution, expressing uncertainty and raising a range of concerns related to ethical issues and privacy. Not only the feeling of being monitored, but also questions related to the collected data and who had access to it, made the older adults worried about their integrity and privacy.

“For there are others who use it maliciously. ... And then there is someone hacking.” (P5).

“What benefit does one get from it? We can never be anonymous…It’s like someone is always watching you.” (P14).

However, a few participants did not have these worries since they compared the system to existing home security systems that they already had.

Further, the participants’ reflections extended beyond their own situations, and they were curious about how homecare managers perceived digital twins and expressed concern for staff integrity and privacy. They also wondered how personnel might feel about the technology, particularly whether it could lead to a sense of being monitored by their managers. There was also concern that digital twins would be used to reduce staffing levels, resulting in fewer homecare staff performing the work, which was expressed by one participant:

“Managers may think that they can use it to reduce staff, so that there is less legwork” (P9).

### 3.4 Opportunities for safety and prevention

Many participants appreciated the idea of digital twins functioning as a real-time surveillance system, especially in situations where their health might suddenly decline. They expressed that having a system capable of detecting changes, such as falls, sudden illness, or health deterioration, could be valuable when they are unable to alert someone themselves.

“When something happens, and you do not alert….if something happens in the head…”. (P11).

One participant who had a stoma bag said:

“…I used to have quite a bit of leakage at night. And then you meant that if they [the staff] could see that it had started leaking, they could be there before I even trigger the alarm. Because maybe I’ve already gotten out of bed, and then I might drop the bag.” (P3).

The participants stated that digital twins may be used as a tool to improve supervision of their situation and to check regularly on them to see if everything is fine. The participants discussed night visits, noting that some felt that the visit contributed to a sense of security, while others mentioned that being woken up could make it hard to fall back asleep. They also reflected on situations where they did not like having visitors. In such cases, some participants felt that digital twins could potentially replace certain physical visits.

Some participants recognized that digital twins could offer more than real-time monitoring; they could see the potential for using digital twins to detect patterns, particularly movement patterns. The participants thought that they could use the information on their movement patterns themselves to become aware of their behaviour, change their behaviour, and/or change the location of their furniture. The movement patterns could also be used by the homecare services to put more home visits in place for preventive purposes, e.g., where the patterns might suggest an increased risk of accidents.

“You can see, perhaps, what my routines look like, what can she do? It may be that they could say, she's being a bit stupid when she does that, and we may have to help her so that it doesn't go wrong in the end because they can't know if I'm perhaps clumsy, turning stupidly in the kitchen, which can be dangerous...” (P8).

“…it actually seems pretty fun, if you get to find out what you’ve done. I might be able to use the information afterwards to become more, more structured, so to speak. And not run around like a headless chicken as it might turn out afterwards, then maybe they will say, then maybe I will realise that I probably shouldn’t be doing so much, it’s just now, it’s what, it’s my experience”. (P4).

### 3.5 Potential effects on daily routines

Several participants saw potential benefits in how digital twins could improve transparency and help highlight inconsistencies in homecare services. For example, some noted that digital twins could reveal when visits occurred outside scheduled timeframes, creating opportunities for improvement.

“Yes, but I think it will come out then so that you can say, we shouldn't have to have it that there should be a two-hour difference at dinner, for example” (P9).

Furthermore, participants imagined that digital twins would be used to optimise time management, allowing more space for meaningful interactions and social visits. At the same time, concerns were expressed that the digital twins would replace social interactions. Some participants connected their experiences of homecare services very strongly to the particular staff who provided the services, and felt it would not be any different with digital twins.

“It depends on which scatter-brained people who come…It is always the person who comes…“. (P1)

“But meeting people….I believe that’s actually better….some things can be addressed with such methods, but not entirely, it should be with people.” (P13)

## 4 Discussion

This study explored older adults’ perspectives on the potential use of digital twins in homecare. The main theme reveals the uncertainties and the possibilities that older adults perceive in relation to digital twins. Participants expressed concerns about the technology, including issues of privacy, integrity, and ethics. At the same time, they saw potential benefits, such as enhanced safety and security, more responsive care tailored to individual needs, and the possibility of more preventive services.

The challenges participants faced in understanding and accepting digital twins align with existing research, which identifies resistance to new technology as a common barrier among older adults, often stemming from the perception that such technology is not relevant or necessary for their everyday lives. One study found that older adults’ willingness to use technology depended on whether they saw themselves in need of it, and that they often viewed technologies as more suitable for others who were perceived as being in greater need.^67^ The challenge of grasping abstract or unfamiliar technologies like digital twins reflects broader concerns about how to contextualize new technology in personally meaningful ways. Research on technology acceptance shows that a technology’s perceived usefulness, together with the perceived ease of use, are the most important factors if a user will accept a technology or not.^68–70^ Acceptance also depends on social influence and facilitating conditions such as organizational and technical infrastructure, which are additionally affected by a person’s age, gender, experience with the technology, and the voluntariness of using a technology.^69^ There is some evidence from a review study that involving older adults in the development of technology may increase their acceptance,^71^ and this may be a worthwhile approach when developing future digital twins. The uncertainties our participants had around ethical aspects of digital twins may also have inhibited their acceptance of the technology.

Older adults receiving homecare express that they are forced to adapt to the homecare services, sacrificing routines, expectations and privacy. They also have to accept being assisted by staff of the opposite sex and adapting to varying staff competencies.^72^ It becomes clear that older adults do not want to be controlled, neither by the homecare services nor by digital twins. Integrity and privacy issues are a common denominator of homecare services and digital twins. They could be compromised by the homecare services, and there was also concern that they might be compromised by digital twins.

Privacy and ethical issues of digital twins, such as the idea of being monitored and integrity issues relating to the collected data, have been discussed in other studies.^4,34,35,73^ In a white paper on the ethical issues that could be faced when dealing with digital twins,^74^ the authors argued that focusing on the performance and productivity of a person might lead to a less inclusive society, and the freedom of choosing whether to use a technology or not will, in the end, be eliminated, forcing people to use the technology. The paper also raised the question of how far humans should be striving to optimize themselves through technology. According to the WHO global strategy on digital health, technologies should, among other factors, be safe, ethical, and person-centred.^75^ While there are many studies discussing and identifying the ethical risks of digital twins,^74,76,77^ we are not aware of any studies that present solutions. It is important that future research moves beyond the identification of ethical risks and begins to explore practical strategies for addressing these ethical challenges. It should also be noted that the integration of digital twins in homecare services must be considered within existing regulatory frameworks, and further research is required to explore the regulatory and policy implications in greater depth. At the same time, it is important to recognise that for some older adults, safety and independence may outweigh privacy concerns. In one study,^78^ older adults with sensors installed in their homes to monitor their movement reported feeling more secure, safe, and independent, and they were generally unconcerned about privacy issues as they prioritized their safety.

Participants recognized the potential of digital twins for highlighting inconsistencies in homecare schedules. Continuity and coordination in care services are important goals in integrated care.^41^ Previous studies have found that having continuity in members of staff, having homecare services that are provided by the same carers, and having enough time during a visit, contribute to safe and secure homecare services.^54,79,80^ Digital twins could support care managers by providing information that makes it easier to address service irregularities and advocate for better continuity and planning.^81^ For example, one study has shown that digital twins can be used to manage staff schedules,^82^ which can improve both staff continuity and the timing of their visits, leading to more satisfaction with homecare. Thus, beyond the ethical concerns, digital twins may also enhance the quality and coordination of care in ways that directly benefit older adults.

The concerns our participants had regarding the costs of digital twins were also found in the Peek et al. (2014) study ^67^where cost was the most important concern for older adults. The cost-effectiveness of digital health interventions has been reviewed, with growing evidence of cost-effectiveness but acknowledgement that comparisons are difficult because of the heterogeneity of such interventions.^83^ Since cost-effectiveness is a critical factor when making policy decisions,^84^ this is an important question to take into consideration for future implementation decisions.

Our participants recognized the potential that digital twins could offer help in real-time, for example when health suddenly declines or accidents occur. They also saw opportunities for more tailored services by detecting individual patterns, which could contribute to more person-centred care. This have been highlighted in previous studies.^21,28,29,74,85^ A digital twin could provide a visual representation of activities, routines, and services that is shared between homecare staff and older adults, thereby fostering a common understanding. This aligns with the principles of person-centred care, emphasizing partnership and shared decision-making.^86^

Our participants realised how digital twins could be used for prediction and, therefore, prevention. This finding is notable, as participants in another study who were more familiar with digital twins than those in our study did not recognize their potential for preventive use.^35^ In another study, participants were not interested in the predictive element of digital twins. The technology was not trusted; they felt it could restrict their lives and make them feel vulnerable. They preferred to live in the ‘here and now’ and for digital twins to be used as mirroring tools to improve their quality of life.^34^

The participants’ concern that technology could replace social interactions in homecare aligns with findings from several studies reporting that older adults were worried that professional caregivers and in-person services could be replaced by technology.^87–90^ This highlights the importance of ensuring that digital innovations, such as digital twins, are implemented in ways that complement rather than substitute human contact, and that the social needs of older adults remain central in homecare planning.

## 5 Strengths and limitations

Collecting data via two methods is a strength of this study. Individual interviews are essential for delving into people’s experiences, as they are a powerful way to gain knowledge about different situations and offer deep insights.^91^ In focus groups, the interaction between group members allows the researcher to gain subtle insights into thoughts and behaviours, and in-depth knowledge of how participants build on each other’s ideas.^92^ The different methods of data collection helped in capturing rich data and increased the study’s credibility. Being interviewed in one’s home environment or being able to elect to be part of a focus group will likely have contributed to the participants’ feeling comfortable and in control, important requisites for being able to express one’s views freely. The size of the focus groups could be questioned since the groups in this study had only three or four participants, but smaller groups can be preferable sometimes when discussing difficult topics, and are less challenging to run than larger groups.^92^

It is not possible to determine why some potential participants and not others were selected by their care managers. At the same time, the recruitment process provided a purposive sample that contained diversity in participants’ homecare needs and use and their living circumstances, contributing to the transferability of the study’s findings.

It might be a limitation that participants had no prior experience with digital twin technology. As a result, their reflections were largely hypothetical, which may have influenced their ability to provide concrete examples of how such technology could be integrated into their daily lives. The fact that participants with cognitive impairment were excluded from the study can also be seen as a limitation since they are an important group of homecare recipients. However, requiring our participants to express their views on an unfamiliar technology was quite cognitively demanding and would perhaps have been too challenging for people with cognitive impairment.

## 6 Conclusions

This study highlights the complexities and challenges older adults face when considering the use of digital twins in homecare services. The study found that older adults have uncertainties and concerns regarding digital twins in homecare, some of which could be due to their lack of awareness of and experience with the technology. The participants did, however, also recognize potential benefits of digital twins, including enhanced safety and a more preventive and tailored homecare responsive to their preferences.

While digital twins have the potential to support person-centred and integrated care, several issues remain to be resolved. Given that the application of digital twins in homecare remains largely unexplored, further research is essential, particularly research that considers older adults’ wishes and needs and actively involves them in the development of future digital twin solutions. Such involvement may help address the valid ethical concerns raised by the participants and clarify the factors that influence older homecare users’ acceptance of digital twins. Access to relevant information, opportunities to interact with the technology, and participation in co-design processes could all shape how older adults assess the value of digital twin solutions.

## Supplemental material

Supplemental material - Unveiling the potential of digital twins in homecare: A reflexive thematic analysis of older adults’ views Supplemental material for Unveiling the potential of digital twins in homecare: A reflexive thematic analysis of older adults’ views by Sandra Saade, Susanna Nordin, Kevin McKee, Marie Elf, Johan Borg in Digital health

Supplemental material - Unveiling the potential of digital twins in homecare: A reflexive thematic analysis of older adults’ views Supplemental material for Unveiling the potential of digital twins in homecare: A reflexive thematic analysis of older adults’ views by Sandra Saade, Susanna Nordin, Kevin McKee, Marie Elf, Johan Borg in Digital health

Supplemental material - Unveiling the potential of digital twins in homecare: A reflexive thematic analysis of older adults’ views Supplemental material for Unveiling the potential of digital twins in homecare: A reflexive thematic analysis of older adults’ views by Sandra Saade, Susanna Nordin, Kevin McKee, Marie Elf, Johan Borg in Digital health

## Data Availability

The data that support the study findings are available from the corresponding author upon reasonable request.*
