# Supplementary material for: Unveiling the potential of digital twins in homecare: A reflexive thematic analysis of older adults’ views
Source: Digit Health. 2026 May 12;12:20552076261450290. doi: 10.1177/20552076261450290 (PMC13172689; doi:10.1177/20552076261450290)
Supplement: Supplemental material - Unveiling the potential of digital twins in homecare: A reflexive thematic analysis of older adults’ views [file sj-pdf-1-dhj-10.1177_20552076261450290.pdf]

Appendix 1. Stage 1-Interview guide

- Can you tell us a bit about yourself? What did you do before retirement?

- How long have you lived here? Who do you live with?

- How long have you had homecare services?

- Tell me about a typical day with homecare services
    - How often and when do you receive homecare services?
    - What services do you receive from the homecare services?
    - How do you experience the homecare services you receive?
        - What is the most important with homecare services?
        - How does it work with the staff?
        - Do you receive services at the time of the day that suits you? /When you need them?

- Do you need other services from the care services than those you already have?
    - What is good? Can you give examples?
    - What could be better? How?
