# Supplementary material for: Unveiling the potential of digital twins in homecare: A reflexive thematic analysis of older adults’ views
Source: Digit Health. 2026 May 12;12:20552076261450290. doi: 10.1177/20552076261450290 (PMC13172689; doi:10.1177/20552076261450290)
Supplement: Supplemental material - Unveiling the potential of digital twins in homecare: A reflexive thematic analysis of older adults’ views [file sj-pdf-2-dhj-10.1177_20552076261450290.pdf]

Appendix 2. Stage 2- Interview guide/focus group discussion

Narrative: I have previously mentioned a digital tool that we in the research project want to develop to improve homecare. We call this digital tool a digital twin.

A digital twin is like a mirror image of something. In this research project, we think it will be a mirror image of what it is like in the home and what the homecare service does. This mirror image is on a computer.

The mirror image shows on a screen what is happening in the home in a simplified way instead of in real life. It can help, for example, homecare managers or planners to understand or support residents and staff in a smart way that was not possible before. No cameras will be used.

- **What is your first thought when you hear this?**
- **What do you think is valuable for a homecare planner or manager to know about you without being with you?**
  - How?
  - Why?

Narrative: The digital twin can look a little different depending on what is being measured in the home. With today's technology, you can measure most things, there are sensors that can show, for example, where you are in the home, if it is cold or hot, if you leave the house, if you lay down, stand or sit, if you use the stove or open the refrigerator, etc... You can also see what times the members of staff come and maybe even what they do. These measurements could also be used to see patterns in movements.

- **Now, based on these examples, is there anything that you think could be useful to measure in your home?**
- **Is there anything that a planner could find out that would make the homecare you receive better?**
  - In what ways?

(Depending on what the participants asked, more information was given to better explain what was meant by the information above.)
